# Intraductal Papillary Neoplasm of the Bile Duct: A Rare Disease and Presentation

**DOI:** 10.7759/cureus.34556

**Published:** 2023-02-02

**Authors:** Alexander Le, Anna Mathew, Ayham Khrais, Irina Khmelnitsky, Sima Vossough

**Affiliations:** 1 Internal Medicine, Rutgers University New Jersey Medical School, Newark, USA; 2 Gastroenterology, Veterans Affairs Medical Center, East Orange, USA; 3 Gastroenterology, Rutgers University New Jersey Medical School, Newark, USA

**Keywords:** cancer, tumor, intraductal papillary neoplasm, biliary disease, bile, neoplasm, cholangiocarcinoma, ipmn, ipnb

## Abstract

Intraductal papillary neoplasm of the bile duct (IPNB) is a rare disease that occurs anywhere along the bile duct. The disease predominantly occurs in Far East Asia and is very rarely diagnosed and documented in western countries. IPNB presents similarly to obstructive biliary pathology; however, patients can be asymptomatic. Surgical resection of IPNB lesions is crucial for patient survival because IPNB is precancerous and can transform into cholangiocarcinoma. Although potentially curative by excision with negative margins, patients who are diagnosed with IPNB need close monitoring for de novo recurrence of IPNB or other pancreatic-biliary neoplasms. In this case, we present an asymptomatic non-Hispanic Caucasian male who was diagnosed with IPNB.

## Introduction

Intraductal papillary neoplasm of the bile duct (IPNB) is a relatively new disease, having been designated by the World Health Organization (WHO) as a bile duct tumor in 2010 [1]. It is defined by the presence of intraluminal papillary tumors with a fibrovascular core anywhere along the bile duct [2]. These tumors are typically multifocal and classified based on subtype and location. The three subtypes of IPNB are gastric, intestinal, and pancreatobiliary, while the three locations are intrahepatic, extrahepatic, and diffuse [3,4]. IPNB is most commonly compared to intraductal papillary mucinous neoplasm (IPMN), however, IPNB is typically pre-malignant/malignant and has lower mucin secretion [5,6].

IPNB is a rare disease most extensively described within the academic literature, and thus presumably by proxy, most likely to affect patient populations of Korea, Japan, and Taiwan. The most common symptoms and clinical laboratory findings at presentation include right hypochondralgia, recurrent episodes of acute cholangitis, and obstructive jaundice with elevated liver enzymes occurring in up to 80% of patients [7]. However, some patients are asymptomatic and diagnosed incidentally. This most commonly occurs in small intrahepatic IPNB. They can resemble simple liver cysts and hemangiomas, making proper identification difficult [8-10].

Laboratory tests are similar to those that present with bile duct obstruction, including elevated bilirubin, alanine aminotransferase (ALT), aspartate aminotransferase (AST), and alkaline phosphatase. Occasionally, carbohydrate antigen 19-9 (CA 19-9), and carcinoembryonic antigen (CEA) may be elevated, but there are no specific laboratory tests to detect IPNB [11]. Ultrasound, computed tomography (CT), magnetic resonance imaging (MRI), cholangiography, and cholangioscopy are the best to assess for bile duct dilation, histopathology of the lesion, and extent of the lesion. The most common means of biopsy include biliary brushing via endoscopic retrograde cholangiopancreatography (ERCP), endoscopic ultrasound (EUS), fine-needle aspiration, or if there is a mass-forming lesion, either EUS or CT-guided core needle biopsy. Overall, IPBNs are unlikely to present with mass-forming lesions, so fine-needle aspiration or brushing is typically performed [12]. When IPNB is found, surgical resection is recommended and has been shown to have a profound impact on survival rates [12]. In this paper, we present an atypical case and presentation of IPNB.

## Case presentation

**Figure 1:**
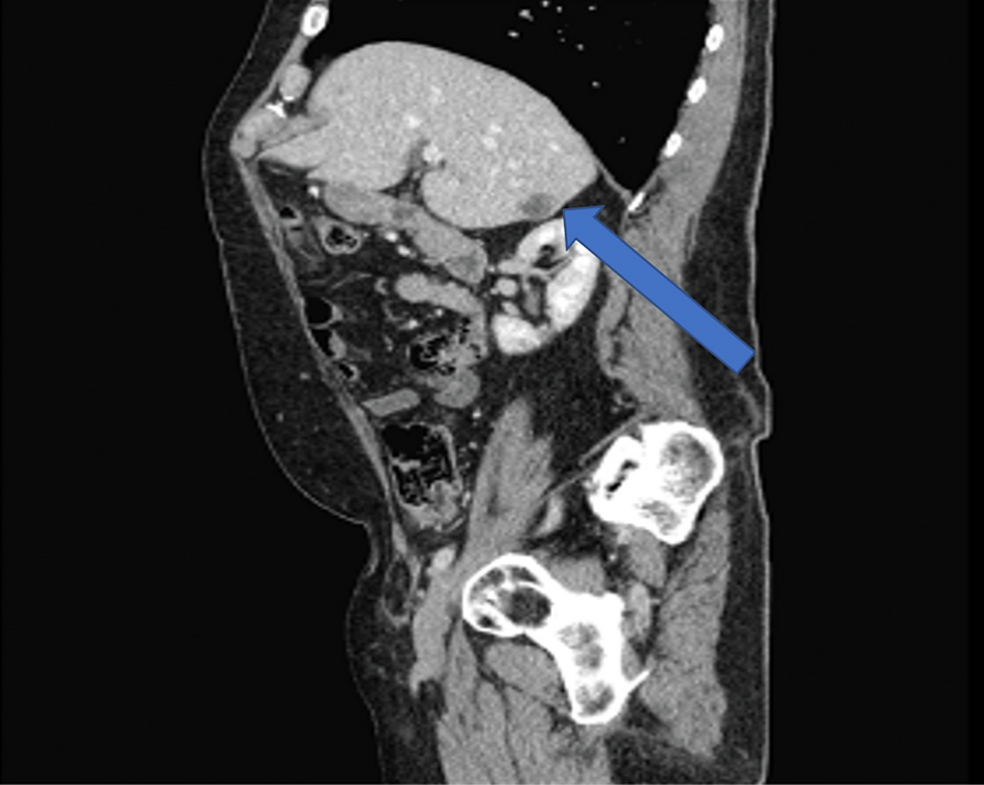
CT abdomen and pelvis triple-sagittal view phase demonstrating a 2.6-centimeter lesion in liver segment 6

**Figure 2:**
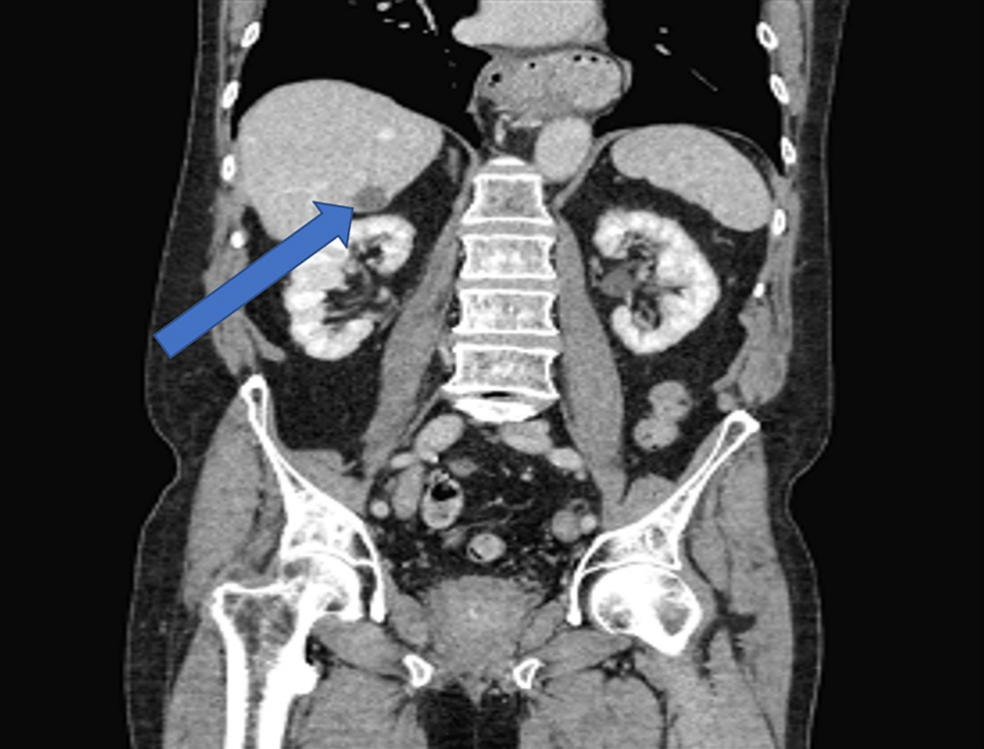
CT abdomen and pelvis triple-coronal view phase demonstrating a 2.6-centimeter lesion in liver segment 6

**Figure 3:**
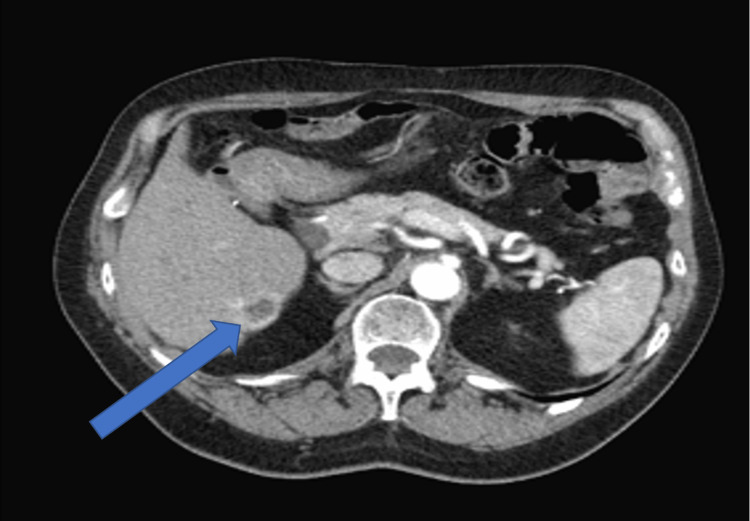
CT abdomen and pelvis triple-transverse view phase demonstrating a 2.6 -centimeter lesion in liver segment 6

**Figure 4:**
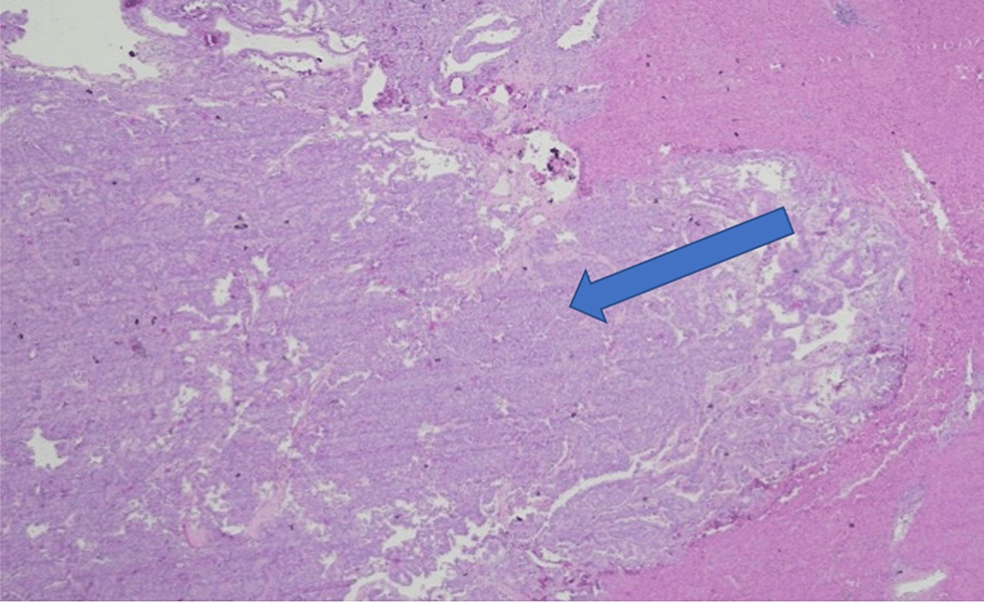
H&E-stained slide showing a neoplasm surrounding by normal liver parenchyma (magnification: 40x)

**Figure 5:**
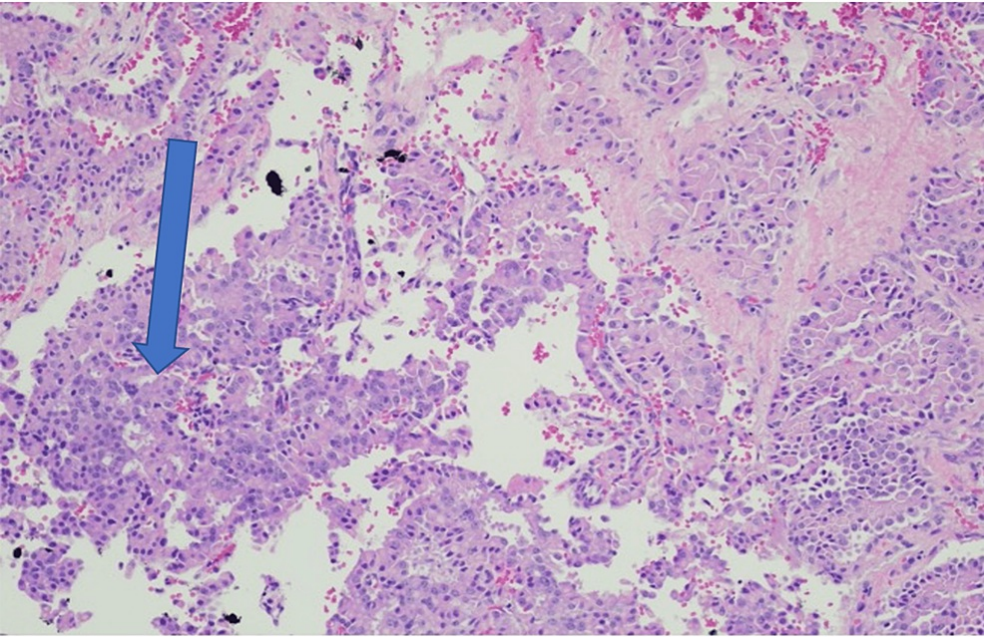
Neoplasm with papillary features

**Figure 6:**
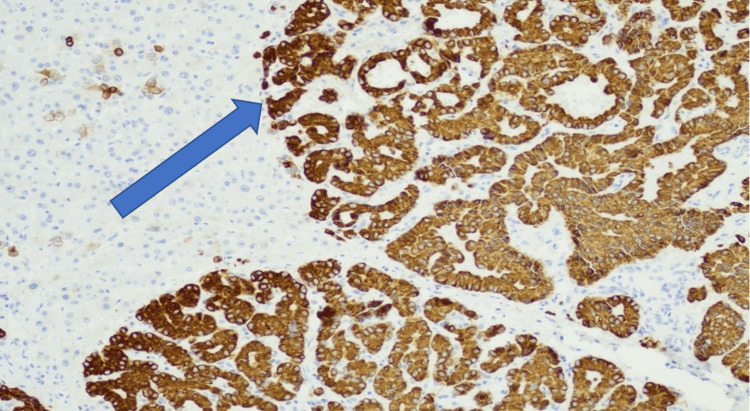
Neoplasm cells positive for CK7

**Figure 7:**
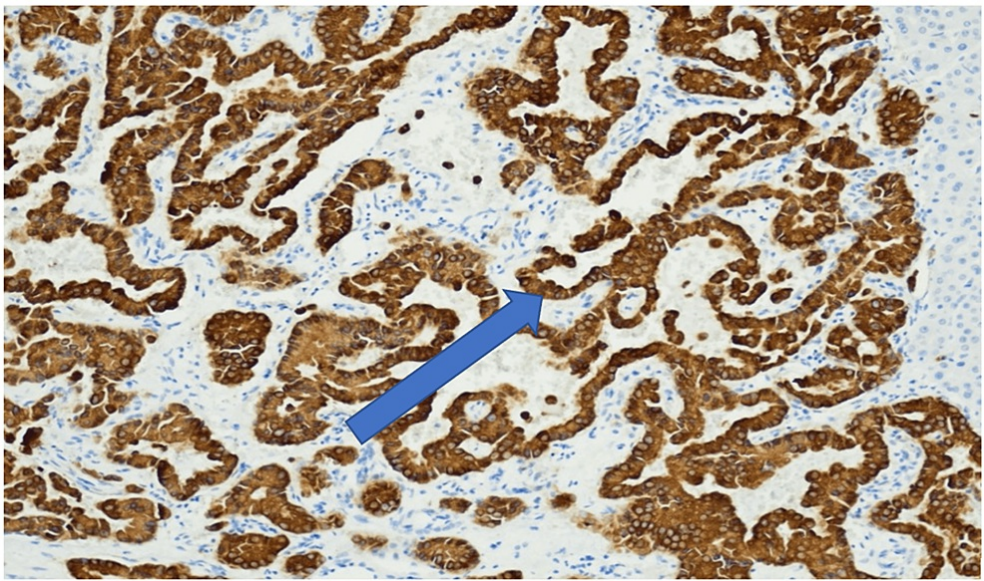
Neoplasm cells positive for CK19

A 73-year-old, non-Hispanic, Caucasian veteran man with a past medical history of multi-lobulated thymic cysts, gangrenous cholecystitis with cholecystectomy, carcinoid duodenal tumors with resection, eosinophilic esophagitis, and hypertension presents with vague abdominal pain. All laboratory tests/values and vitals, including bilirubin, alkaline phosphatase, AST/ALT, lipase, and tumor markers including AFP, CEA, and CA 19-9 were within normal limits. He was incidentally found to have a hypoattenuating but vascular segment 6 liver lesion on CT abdomen and pelvis with contrast, which was initially thought to be a hemangioma. Follow-up triple-phase CT one month later showed vascular enhancement of the lesion and enlargement from 0.8 cm to 2.6 centimeters (Figures 1-3). MRI and magnetic resonance cholangiopancreatography (MRCP) were deferred due to the patient's severe claustrophobia. The patient underwent an image-guided (IR) liver biopsy, which showed bland glands with oncocytic cytoplasm immunotype (pankeratin+, cytokeratin 7+ (CK7+), CDX2+, cytokeratin 20- (CK20-), and hepatocyte-specific antigen- (HSA-) consistent with the pancreatic-biliary origin, concerning for a neoplasm (Figures 4-7). Two months later, the patient underwent a segment 6 hepatectomy with surgical pathology showing intraductal papillary neoplasm of the intrahepatic bile ducts (IPN-B) with high-grade dysplasia.

## Discussion

Several aspects of the patient's history contributed to a delayed diagnosis. First, this patient’s history of a gangrenous gallbladder with cholecystectomy 20 years prior, which necessitated urgent cholecystectomy and post-surgical recovery may have delayed the patient’s subsequent diagnostic workup of IPNB. Additionally, his cholecystectomy resulted in the patient having a baseline dilated common bile duct of ~1 cm. Specifically, post-cholecystectomy bile duct dilatation of up to 10 mm can be considered within the normal range and presents asymptomatically, as in our patient [13]. Detection of duct dilation can often aid in IPNB diagnosis, as IPNB commonly shows mucin hypersecretion [14]. However, with our patient’s history and existing baseline dilation, determining the cause of this dilation (mucin production or cholecystectomy) further complicated the diagnosis. Furthermore, this patient remained clinically asymptomatic. Thus, a liver biopsy was only considered after the rapid growth of a liver mass.

The most common surgical intervention is a pancreaticoduodenectomy, but only about 15% of patients with IPNB undergo segmental liver resection [15], as has been the case for our patient.

In this case, the tumor was limited to the liver, and negative margins were confirmed on pathology. IPNB has been well-documented to show superficial spread within and around the biliary tract [16]. Inadequate resection of areas showing dysplastic and atypical epithelium has been shown to decrease survival compared to those with negative margins [17].

When surgery cannot be performed, palliative care treatment options include chemotherapy, biliary drainage, and laser ablation [2]. Overall, the median survival rate is 62 months after initial diagnosis and is affected by cellular atypia, lymph node metastasis, and depth of invasion of cancer [18].

About 13-29% of patients diagnosed with IPNB will have recurrence after surgical resection. This number increases to 47-62% once patients are diagnosed with invasive IPNB [19,20]. This led to the theory of multicentricity - IPNB originating in multiple locations along the biliary tract [20]. If this is true, even surgical resection with negative margins may not be enough for curative treatment. Thus, close follow-up is needed. In the case we present, we proposed an MRI abdomen every three months for the patient, with surveillance EUS and biliary brushing. Additionally, since post-surgical biliary sampling can be quite challenging to interpret from a cytopathology standpoint, there are now new clinical molecular diagnostic modalities to further parse atypical lesions that may or may not require additional surgical interventions such as next-generation sequencing of cyst fluid or cell block brushing [21].

## Conclusions

INPB is a rare biliary tumor with intraductal growth rather than frank invasion and, therefore, a much better prognosis than conventional cholangiocarcinoma. It can present insidiously and asymptomatically in patients. Rapidly growing hepatic and biliary masses should be worked up with INPB in the differential diagnosis. Resection, histopathologic examination, and close follow-up are important favorable factors for long-term survival.
